# Supplementary material for: Salivary Biomarkers and Their Correlation with Pain and Stress in Patients with Burning Mouth Syndrome
Source: J Clin Med. 2020 Mar 28;9(4):929. doi: 10.3390/jcm9040929 (PMC7230786; doi:10.3390/jcm9040929)
Supplement: Supplementary file 1 [file jcm-09-00929-s001.pdf]

Supplementary Materials

Table S1 Median and interquartile range data of salivary markers in healthy controls (n = 31) and patients suffering from BMS (n = 51).

Marker	Controls		BMS		*P*
	Median	25-75%	Median	25-75%	
**Absolute Values**					
Salivary flow rate, mL/5min	1.27	0.85-2.22	1.81	1.25-2.99	**0.038**
Total proteins, mg/dL	122.09	94.9-211.23	157.10	85.89-275.91	0.238
Uric Acid, mg/dL	3.73	2.83-5.27	3.24	1.88-5.88	0.576
sAA, IU/L	92680	37270-144180	141340	83815-231445	**0.002**
IgA, mg/dL	28.65	11.89-51.21	51.12	24.95-105.87	**0.012**
FRAP, mmol/L	0.61	0.48-0.87	0.55	0.33-0.89	0.549
Hp, ng/mL	1366.64	1023.6-2598	1593.12	1072.72-2605.6	0.518
CRP, ng/mL	25.01	10.93-54.07	17.93	8.52-66.46	0.534
a1AT, ng/mL	1.31	0.44-4.99	1.31	0.7-2.81	0.825
PEDF, ng/mL	5.25	1.95-13.70	8.57	3.29-16.04	0.156
SAP, ng/mL	28.68	15.12-34.85	20.05	9.7-38.4	0.432
MIP4, ng/mL	0.06	0.02-0.08	0.05	0.03-0.11	0.565
CC4, ng/mL	11.11	4.26-20.79	10.15	6.23-22.01	0.655
**Corrected by the flow**					
Uric Acid, ng/min	10.23	1.26-17.95	14.03	8.05-19.51	0.264
sAA, IU/min	17.71	5.33-42.61	61.75	32.43-117.03	**<0.001**
IgA, ng/min	56.08	30.76-141.39	176.00	97.79-364.26	**<0.001**
FRAP, mmol/min	0.17	0.09-0.28	0.22	0.14-0.31	0.185
Hp, ng/min	471.22	138.26-997.52	611.5	336.04-1271.51	0.139
CRP, ng/min	5.61	1.77-14.23	10.7	3.55-19.11	0.189
a1AT, ng/min	0.28	0.12-1.13	0.47	0.31-1.10	0.063
PEDF, ng/min	0.82	0.08 – 2.16	0.98	0.21-2.33	0.259
SAP, ng/min	6.71	2.49-14.41	7.06	4.05-16.52	0.236
MIP4, pg/min	11.56	5.88-20.51	24.59	12.71-42.95	**<0.001**
CC4, ng/min	2.94	1.44-5.06	5.17	1.79-8.03	0.090
**Corrected by the TP**					
Uric acid, mg/g	32.69	17.37-41.49	21.24	13.84-30.81	**0.043**
sAA, IU/g	65695.93	29123.64-92371.35	104220.58	67602.09-144772.25	**0.002**
IgA, mg/g	229	90.07-352.6	296.09	179.36-481.09	0.094
FRAP, mmol/g	0.50	0.33-0.66	0.33	0.26-0.50	**0.019**
Hp, mg/g	1231.16	586.04-1942.86	1034.73	688.04-1461.08	0.758
CRP, mg/g	20.56	8.19-32.98	13.86	7.59-22.6	0.169
a1AT, mg/g	1.17	0.36-2.79	0.91	0.38-1.82	0.383
PEDF, mg/g	4.78	1.55-8.57	6.01	3.39-9.08	0.234
SAP, mg/g	23.42	10.32-29.43	13.78	8.14-19.98	0.068
MIP4, mg/g	0.04	0.02-0.06	0.04	0.03-0.06	0.926
CC4, mg/g	7.73	2.75-17.42	7.66	3.53-13.58	0.845

sAA, salivary alpha-amylase; IgA, immunoglobulin A; Hp, haptoglobin; CRP, C-reactive protein; a1AT, α1-Antitrypsin; PEDF, Pigment epithelium-derived factor; SAP, serum amyloid P; MIP4, Macrophage Inflammatory Protein-4; CC4, Complement C4; TP, total proteins. Data in bold highlight statistical significance.

Table S2. Partial correlation adjusted for age and sex.

	**VAS**<br>**r (*P*)**	**OHIP14**<br>**r (*P*)**	**HAD-A**<br>**r (*P*)**	**HAD-D**<br>**r (*P*)**	**Caries**<br>**r (*P*)**	**Missing teeth**<br>**r (*P*)**
**Absolute Values**						
Salivary flow rate, mL/5min	0.233 (0.054)	0.21 (0.083)	0.035 (0.774)	-0.100 (0.415)	-0.039 (0.749)	0.019 (0.878)
TP, mg/dL	**0.241 (0.046)**	0.15 (0.218)	**0.251 (0.037)**	0.189 (0.119)	-0.007 (0.952)	-0.086 (0.485)
Uric Acid, mg/dL	0.068 (0.581)	-0.103 (0.398)	-0.014 (0.912)	0.078 (0.523)	0.028 (0.819)	0.006 (0.960)
sAA, IU/L	**0.260 (0.031)**	0.111 (0.363)	0.125 (0.305)	-0.059 (0.632)	-0.064 (0.602)	-0.049 (0.687)
IgA, mg/dL	**0.334 (0.005)**	**0.273 (0.023)**	**0.302 (0.012)**	**0.261 (0.030)**	-0.03 (0.805)	-0.012 (0.923)
FRAP, mmol/L	0.088 (0.472)	-0.085 (0.487)	-0.035 (0.775)	0.082 (0.501)	0.03 (0.808)	0.002 (0.989)
Hp, ng/mL	0.079 (0.518)	-0.001 (0.993)	0.057 (0.639)	0.044 (0.717)	0.02 (0.872)	0.007 (0.955)
CRP, ng/mL	0.04 (0.746)	-0.062 (0.613)	-0.075 (0.541)	-0.009 (0.943)	0.044 (0.72)	0.018 (0.883)
a1AT, ng/mL	-0.073 (0.55)	-0.045 (0.716)	-0.11 (0.37)	-0.146 (0.231)	0.071 (0.56)	-0.281 (0.019)
PEDF, ng/mL	0.127 (0.297)	0.222 (0.067)	**0.351 (0.003)**	**0.363 (0.002)**	0.006 (0.961)	-0.081 (0.506)
SAP, ng/mL	0.073 (0.553)	0.011 (0.931)	-0.025 (0.841)	-0.11 (0.370)	-0.028 (0.82)	-0.034 (0.783)
MIP4, ng/mL	0.11 (0.368)	0.205 (0.091)	**0.267 (0.026)**	**0.271 (0.024)**	-0.024 (0.842)	-0.124 (0.311)
CC4, ng/mL	-0.009 (0.941)	-0.023 (0.851)	-0.034 (0.782)	0.099 (0.420)	0.138 (0.257)	0.083 (0.495)
**Corrected by the flow**						
Uric Acid, mg/min	0.113 (0.357)	-0.157 (0.199)	-0.099 (0.419)	-0.106 (0.384)	-0.046 (0.71)	-0.032 (0.797)
sAA, IU/min	**0.269 (0.025)**	0.14 (0.251)	0.102 (0.402)	-0.047 (0.701)	-0.099 (0.418)	-0.011 (0.926)
IgA, mg/min	**0.367 (0.002)**	**0.313 (0.009)**	**0.338 (0.004)**	0.195 (0.108)	-0.055 (0.653)	-0.023 (0.851)
FRAP, mol/min	0.204 (0.092)	-0.017 (0.89)	-0.112 (0.36)	-0.119 (0.331)	-0.042 (0.733)	-0.05 (0.682)
Hp, ng/min	0.179 (0.14)	0.173 (0.156)	0.02 (0.871)	-0.043 (0.723)	-0.044 (0.718)	0.013 (0.917)
CRP, ng/min	-0.044 (0.72)	-0.081 (0.508)	-0.152 (0.213)	-0.161 (0.185)	-0.006 (0.961)	0.001 (0.992)
a1AT, ng/min	0.111 (0.364)	0.135 (0.269)	-0.106 (0.386)	-0.154 (0.207)	0.043 (0.725)	-0.16 (0.188)
PEDF, ng/min	0.122 (0.318)	0.198 (0.104)	**0.297 (0.013)**	**0.386 (0.001)**	0.01 (0.937)	-0.05 (0.682)
SAP, ng/min	0.072 (0.555)	0.01 (0.937)	-0.026 (0.834)	-0.109 (0.371)	-0.028 (0.819)	-0.032 (0.792)
MIP4, ng/min	0.116 (0.342)	0.22 (0.069)	**0.264 (0.028)**	**0.269 (0.026)**	-0.009 (0.938)	-0.127 (0.299)
CC4, ng/min	0.167 (0.171)	0.044 (0.718)	-0.025 (0.841)	-0.068 (0.58)	0.059 (0.633)	-0.052 (0.669)
**Corrected by the TP**						
Uric acid, mg/g	0.161 (0.171)	0.136 (0.249)	0.153 (0.193)	0.123 (0.297)	-0.021 (0.859)	0.086 (0.468)
sAA, IU/g	-0.183 (0.119)	**-0.242 (0.038)**	-0.204 (0.082)	-0.109 (0.356)	0.013 (0.910)	0.022 (0.850)
IgA, mg/g	0.183 (0.118)	0.117 (0.321)	0.171 (0.145)	0.038 (0.750)	-0.018 (0.879)	-0.006 (0.961)
FRAP, mmol/g	-0.188 (0.109)	-0.222 (0.058)	**-0.250 (0.032)**	-0.122 (0.299)	0.025 (0.832)	0.005 (0.963)
Hp, mg/g	-0.063 (0.593)	-0.049 (0.676)	-0.098 (0.408)	-0.09 (0.446)	-0.109 (0.356)	-0.001 (0.992)
CRP, mg/g	-0.15 (0.201)	-0.157 (0.182)	**-0.243 (0.037)**	-0.17 (0.147)	0.092 (0.437)	0.007 (0.950)
a1AT, mg/g	-0.203 (0.083)	-0.165 (0.161)	-0.208 (0.075)	-0.187 (0.111)	0.074 (0.531)	-0.155 (0.187)
PEDF, mg/g	-0.05 (0.675)	0.043 (0.719)	0.153 (0.194)	0.159 (0.177)	0.064 (0.588)	0.032 (0.787)
SAP, mg/g	0.069 (0.558)	0.006 (0.961)	-0.026 (0.823)	-0.099 (0.401)	-0.025 (0.831)	-0.022 (0.853)
MIP4, mg/g	-0.021 (0.857)	0.086 (0.464)	0.154 (0.189)	0.165 (0.160)	-0.012 (0.917)	-0.035 (0.768)
CC4, mg/g	-0.129 (0.272)	-0.115 (0.330)	-0.093 (0.433)	-0.042 (0.721)	0.164 (0.162)	0.002 (0.987)

sAA, salivary alpha-amylase; IgA, immunoglobulin A; Hp, haptoglobin; CRP, C-reactive protein; a1AT, α1-Antitrypsin; PEDF, Pigment epithelium-derived factor; SAP, serum amyloid P; MIP4, Macrophage Inflammatory Protein-4; CC4, Complement C4. Data in bold highlight statistical significance.
